# Effect of Novel All-in-One Root Canal Irrigants on Dislodgement Resistance of Epoxy Resin and Hydraulic Calcium Silicate Sealers: An *Ex vivo* Study

**DOI:** 10.1055/s-0045-1811574

**Published:** 2025-09-04

**Authors:** Shreeraksha Kamath, Rajkumar Narkedamalli, Krishna Prasad Shetty, Nidambur Vasudev Ballal

**Affiliations:** 1 Department of Conservative Dentistry and Endodonitcs, Manipal College of Dental Sciences, Manipal Academy of Higher Education, Manipal, India; 2 Department of Clinical Science, Centre of Medical and Bio-allied Health Science Research, College of Dentistry, Ajman University, Al-Jurf Ajman, United Arab Emirates

**Keywords:** adhesion, Dual Rinse HEDP, irrigation, root canal sealers, Triton

## Abstract

**Objective:**

To investigate the dislodgement resistance of AH Plus and CeraSeal sealers to root dentin conditioned with Dual Rinse 1-hydroxyethylidene-1,1-diphosphonic acid (HEDP) (DR HEDP) or Triton and to correlate their effects on the organic and inorganic content of the root dentin treated with test irrigants.

**Materials and Methods:**

Sixty single-rooted extracted human teeth were divided into two groups and irrigated with DR HEDP or Triton. These samples were further divided into two subgroups to study the dislodgement resistance of AH Plus and CeraSeal sealers. Middle third root sections were obtained, filled with AH Plus or CeraSeal sealers, and were subject to push-out bond strength (POBS) analysis. Fourier-transform infrared (FTIR) spectra of root dentin treated with test irrigants were obtained to analyze the changes in organic and inorganic content.

**Statistical Analysis:**

POBS and FTIR values were normally distributed and hence the average values were contrasted among the groups using one-way analysis of variance with the post hoc Tukey's honest significant difference test.

**Results:**

DR HEDP exhibited the highest dislodgement resistance of both CeraSeal and AH Plus sealers to root dentin in contrast to Triton and saline. Saline demonstrated the lowest dislodgement resistance with both the sealers tested. Posttreatment amide III: phosphate ratio was increased with saline compared with DR HEDP or Triton. Posttreatment carbonate: phosphate ratio was increased with DR HEDP compared with Triton or saline.

**Conclusion:**

Root conditioning with DR HEDP prior to sealer placement was beneficial for the adhesion of both AH Plus and CeraSeal sealers.

## Introduction

Microorganisms and their metabolic products are the decisive causal factors for the development and persistence of pulpal and periradicular pathologies. Hence, the total elimination of microorganisms and their metabolic products from the infected canals is the key to the success of root canal treatment. 1 This may be accomplished by mechanical instrumentation, irrigation with disinfectant solutions, and the application of interappointment intracanal medicaments. Previous literature has demonstrated that mechanical instrumentation alone cannot entirely eliminate microorganisms from the complex inaccessible regions of the root canal system, such as isthmuses, fins, cul-de-sacs, lateral canals, or deep dentinal tubules, resulting in the retention of biofilms *in situ* . 2 Subsequently, irrigation plays a vital part in the success of root canal treatment by enabling the removal of bacterial biofilms and infected tissue debris. Sodium hypochlorite (NaOCl) is the frequently used root canal irrigant 3 due to its unique characteristic of dissolution of soft tissue, 4 biofilms, and their matrix. 5 Traditionally, NaOCl is complemented with a chelating agent such as EDTA, to eliminate the smear layer generated while mechanically instrumenting the root canals. 6 Recently, a product branded as Dual Rinse 1-hydroxyethylidene-1,1-diphosphonic acid (HEDP) (DR HEDP; Medcem GmbH, Weinfelden, Switzerland) is commercially obtainable, which contains etidronate or HEDP. It is dispensed as a capsule containing 0.9 g of etidronate powder, which must be mixed instantly prior to treatment with 10 mL of a NaOCl solution (0.5–5.25%) to yield an all-in-one irrigant. 7 This all-in-one irrigant allows clinicians to apply DR HEDP in a “continuous chelation” approach during chemomechanical preparation of the root canal system. 8 The apparent advantage of the “continuous chelation” method compared with consecutive utilization of NaOCl and a chelating agent is that only one irrigant is necessary for the entire chemomechanical preparation, which significantly cuts irrigation time. Earlier literature demonstrated that DR HEDP exhibits efficacy comparable to that of NaOCl in its antibacterial and tissue-dissolving efficacy, 4 9 is nontoxic, 10 and enhances the bonding of sealers and cements to radicular dentin. 11 12

Triton is another recently introduced all-in-one endodontic irrigation solution (Brasseler, Savannah, Georgia, United States). It has two components (Part A and Part B), which are meticulously blended via an automix cap to yield the finished solution. Its formulation contains 2-phophonobutane-1,2,4-tricarboxylic acid (PBTC; <15%), citric acid (<2%), NaOCl (<10%), sulfonate and disulfonate surfactants, polyacrylate, ethoxylated alcohol, sodium tripolyphosphate, modified polyethylene glycol, sodium lauryl sulfate, sodium hydroxide, and amine oxide. Studies lately have established its effectiveness in eliminating debris and smear layer, as well as its antibacterial properties. 13 14 15

The complete sealing of the root canal space after disinfection is an essential phase in root canal treatment to avert the ingress of bacteria from the oral environment. This can be achieved through three-dimensional obturation using gutta-percha and a sealer. 16 The bond strength of a sealer to root dentin is a crucial characteristic for preserving the integrity of the filling material. 17 Epoxy resin-based sealers such as AH Plus (Dentsply DeTrey, Konstanz, Germany) have been extensively utilized in endodontics, attributing to their suitable physical properties, decreased solubility, good sealability, superior adhesion to radicular dentin, and satisfactory biological activity. 18

Recently, hydraulic calcium silicate (CaSi)-based sealers have attained growing popularity in endodontics due to their higher biocompatibility, bioactivity, and their capability to set in a moist environment. 19

CeraSeal is a premixed CaSi-based bioceramic sealer, which has been shown to release calcium ions and alkalize the surrounding environment. It has been found to have inferior radiopacity and setting time in contrast to AH Plus, while having similar flowability. 20 21 22

A recent study reported that roots obturated with the single cone technique and CeraSeal premixed bioceramic sealer demonstrated elevated survival and healing rates subsequent to 36 months. 23

Previous investigations have revealed that there are several methods by which modern root canal sealers adhere to the treated root canal walls. 11 24 25 Epoxy resin sealers adhere to exposed collagen fibrils in the root dentin. 26 However, hydraulic CaSi sealers form a mineral infiltration zone with the root dentin surface, leading to a chemical bond with calcium phosphate, integrated with micromechanical interlocking by the creation of cement tags within the dentinal tubules. 24 27 Therefore, advisable to align the irrigation protocol with the utilized sealer materials. 24 Previous studies have demonstrated the effect of various root canal irrigants on the bond strength of epoxy resin and CaSi sealers to radicular dentin. 11 28 29

Although Triton has been evaluated for its efficiency in the eradication of smear layer, tissue dissolution, and antimicrobial activity, its effect on the bonding of sealers to radicular dentin has not been investigated. Besides this, no comparative investigation has assessed the effect of all-in-one irrigants DR HEDP versus Triton on the push-out bond strength (POBS) of epoxy resin and CaSi sealers to root dentin. Hence, the objective of the present ex vivo study was to evaluate the POBS of AH Plus and CeraSeal sealers to root canal dentin conditioned with DR HEDP or Triton, and evaluate the effect of these irrigants on the organic and inorganic content of the root canal dentin. The null hypothesis tested was that DR HEDP and Triton have the same impact on the POBS of AH Plus and CeraSeal sealers to radicular dentin.

## Materials and Methods

### Ethical Clearance

The utilization of human-extracted teeth for the studies conducted in this work received approval from the Institutional Review Board of the first author's university (36/2025).

### Sample Size Estimation

The sample size in the current study was based on the influence of NaOCl with HEDP on the bond strength of an epoxy or MTA-based sealer to root dentin. 1 The G*Power software (Heinrich Heine University, Dusseldorf, Germany) was used for the estimation of sample size. With a 95% confidence level, 80% power, a standard deviation of 0.6, and a mean difference of 0.6, the minimum sample size was estimated to be 10 per group. For Fourier-transform infrared (FTIR) analysis, five samples were used per group.

### Sample Preparation

Sixty noncarious human mandibular single-rooted premolar teeth, extracted for orthodontic or periodontal purposes, were chosen. Teeth with straight roots and a curvature of less than 30 degrees were involved. 30 A radiograph was recorded for every tooth from mesial and distal direction, and the tooth was assessed under a surgical microscope (EXTARO 300, Carl Zeiss Meditec AG, Jena, Germany) to verify the presence of a single-rooted canal with mature apices, and the absence of intraradicular resorption, calcification, or root canal fillings. The teeth were cleaned with an ultrasonic scaler and kept in an aqueous solution of 0.2% sodium azide (MilliporeSigma, Burlington, Massachusetts, United States) at 4°C until use. Every single tooth was then decoronated using a diamond disk (Horico, Berlin, Germany), and working length was estimated by introducing a size 10 K file (Mani Inc., Tochigi Ken, Japan) into each root canal until it was just observable at the apical foramen, then deducting 1 mm from the recorded length. Apices of all the teeth were closed with sticky wax, and the roots were fixed in polyvinylsiloxane impression material (3M ESPE, St. Paul, Minnesota, United States) to avoid the flow of irrigants through the apex and to ensure an effective reverse flow of the irrigant, thus mimicking a closed-end system. 31 The root segments were arbitrarily assigned to two experimental groups and a control group ( *n* = 20) with the help of a random sequence generator ( http://www.random.org ). Root canal instrumentation was then performed till the working length using the ProTaper universal nickel–titanium rotary system (Dentsply Sirona Endodontics, Ballaigues, Switzerland) per the manufacturer's instructions, up to a size of F3. The canal taper was then eliminated with the help of Peeso reamer size 1 to 2 (Mani Inc.). The three groups assessed were:

1. *DR HEDP* : 2 mL of DR HEDP (Medcem GmbH) for 1 minute between each transition of instruments and a final flush using 5 mL of DR HEDP for 1 minute.
2. *Triton* : 2 mL of Triton (Brasseler [LOT: 20222938]) for 1 minute between each transition of instruments, and a final flush using 5 mL of Triton for 1 minute.
3. *Saline (control)* : 2 mL of saline for 1 minute between each transition of instruments and a final flush using 5 mL of saline for 1 minute.

In the DR HEDP group, one capsule of DR HEDP (0.9 g) was dissolved in 10 mL of the 3% NaOCl (Vista Dental Products, Racine, Wisconsin, United States) solution. In the Triton group, using the automix technique, the solutions from both components (A and B) were aspirated using a syringe as recommended by the manufacturer and were used immediately. All irrigants were delivered using a 30-gauge stainless steel side-vented needle (Appli-Vac, Vista Dental Products), with the needle tip placed 1 mm short of the working length in each canal. Upon performing the designated irrigation regimen, each canal was flushed with 5 mL of distilled water for 1 minute to eradicate any chemical precipitates from the root canal system. 32

### Push-out Bond Strength Analysis

Subsequent to the final irrigation protocol, the root canals were desiccated utilizing paper points (Dentsply Sirona Endodontics). Samples in each group were then subdivided into two groups ( *n* = 10, each) as per the sealers used, AH Plus (Dentsply DeTrey, Konstanz, Germany) versus CeraSeal (Meta Biomed Co., Ltd., Cheongju-si, Korea). The teeth were horizontally split at the middle third using an Isomet saw (Isomet 1000, Buehler Ltd, Lake Bluff, Illinois, United States) under continuous water cooling to attain a dentin slice of 2.0 ± 0.1 mm thickness. Sealers were then mixed as per the manufacturer's instructions and filled into the root canal space. Samples were placed in 100% humidity for 48 hours to confirm the thorough setting of the sealers.

Every sectioned tooth sample that was filled with sealer was then subjected to POBS estimation. The diameter of the root canal and the thickness of each slice were measured with a digital calliper. The push-out test was conducted using a universal testing machine (Instron, Massachusetts, United States). The force was applied at a crosshead speed of 1 mm/min using stainless steel plungers with a diameter of 0.7 mm, arranged to ensure contact solely with the filling material. The maximum force ( *F* ) applied at bond failure was documented in Newtons. The POBS was computed in MPa via the formula: POBS (MPa) = Force (N)/adhesion surface area (mm ^2^ ).

The adhesion surface area was estimated by the following equation:

Adhesion surface area (mm ^2^ ) = 2 × π × *r* × *h* , where *r* is the root canal radius, π is the constant 3.14, and *h* is the thickness of the root slice.

### Fractographic Analysis

All samples from each group were subjected to stereomicroscopic analysis (Zeiss, Thornwood, New York, United States) at ×40 magnification for the assessment of bond failures. The modes of bond failures were sorted as:

1. *Adhesive failure* : between the root canal dentin wall and the sealer interface.
2. *Cohesive failure* : within the sealers.
3. *Mixed failure* : a combination of both adhesive and cohesive failures.

### Attenuated Total Reflectance–Fourier-Transform Infrared Analysis

A total of 15 root dentin blocks measuring 1 cm ×1 cm were prepared from eight extracted human maxillary central incisors. The root canal dentin surface of each sample was serially wet polished by employing 800 to 1,200 grit abrasives with a single disc table-top polisher (Bainpot VT, Guadalajara, Mexico). This procedure rendered a smooth surface that could favor the absorbance of infrared radiation. This was followed by immersion of the samples into distilled water under an ultrasonic bath (1 minute) for removal of the residual material after polishing. The samples were blotted dry using absorbent paper points (Dentsply Sirona Endodontics) to mimic the clinical environment and reproduce the tissue characteristics, thereby avoiding excessive dehydration. The samples were then equally categorized into three groups ( *n* = 5), similar to the irrigation protocol employed for POBS analysis. Pretreatment compositional analysis of amide III, phosphate, and carbonate infrared bands was performed using an FTIR spectrophotometer equipped with a diamond ATR setup (JASCO, Deutschland GmbH, Pfungstadt, Germany). Then, the samples were treated with irrigating agents similar to POBS analysis. Samples were positioned in test tubes containing 10 mL of the experimental irrigating agents for 30 minutes under ultrasonic agitation. After the stipulated time period, the remnants of irrigating solutions were removed by immersing the samples in distilled water (10 mL) for 10 minutes, followed by drying using absorbent points (Dentsply Sirona Endodontics). Posttreatment infrared spectrum areas of the respective bands were determined using baseline tracing. Spectra were obtained between 400 and 4,000/cm at 4/cm resolution by using 64 scans per measurement. The alterations seen in the organic and inorganic components in the dentin were used in calculating the amide III:phosphate and carbonate:phosphate ratios, respectively.

### Statistical Analysis

Data were statistically analyzed using SPSS Statistics Version 25.0 software (IBM Corp, Armonk, New York, United States). The Kolmogorov–Smirnov's test was used to assess the normality of the values. POBS and FTIR values were evenly distributed, and hence, the mean values were contrasted among the groups utilizing one-way analysis of variance followed by the post hoc Tukey's honest significant difference test. The α-type error was established at 5%.

## Results

### Push-out Bond Strength Analysis

**Fig. 1:**
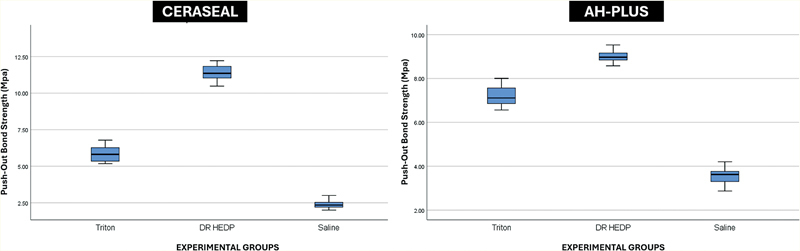
Box plots with error bars representing maximum and minimum POBS values (MPa) obtained with two different sealers applied to the root canal dentin conditioned with different irrigating solutions. Dual Rinse HEDP demonstrated the highest POBS followed by Triton and saline in both CeraSeal and AH Plus sealers ( *p* < 0.001).

DR HEDP demonstrated the highest POBS with respect to both AH Plus (8.98 ± 0.2 MPa) and CeraSeal (11.36 ± 0.5 MPa) sealers, while Triton demonstrated higher POBS compared with saline for both AH Plus (7.20 ± 0.4 MPa) and CeraSeal (5.85 ± 0.5 MPa) sealers ( *p* < 0.001). The lowest POBS was observed in the saline group for both AH Plus (3.56 ± 0.3 MPa) and CeraSeal (2.39 ± 0.3 MPa) sealers ( Fig. 1 ).

### Fractographic Analysis

**Fig. 2:**
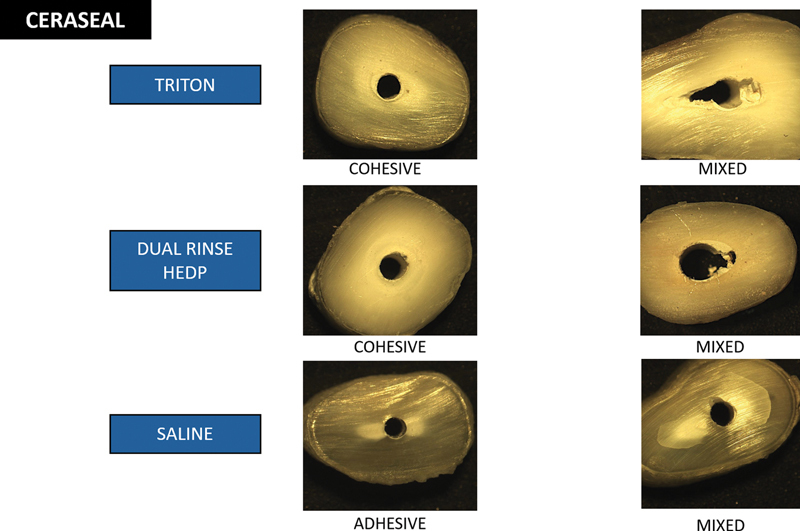
Stereomicroscopic images of bond failures of CeraSeal sealer samples treated with experimental irrigating agents.

**Fig. 3:**
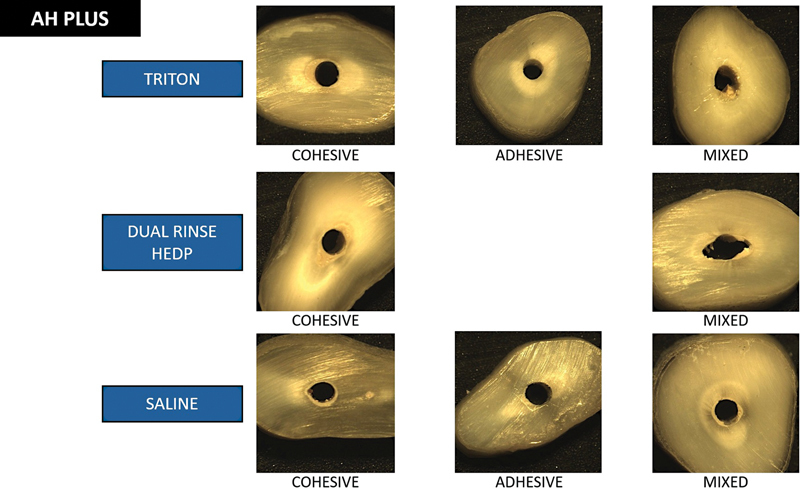
Stereomicroscopic images of bond failures of the AH Plus sealer samples treated with experimental irrigating agents.

For the AH Plus sealer, the Triton group had 50% cohesive, 30% adhesive, and 20% mixed failures, while the DR HEDP group demonstrated 70% cohesive failures and 30% mixed failures. In the saline group, there were 60% adhesive failures, 20% cohesive failures, and 20% mixed failures. For the CeraSeal sealer, the Triton group had 60% mixed and 40% cohesive failures, while the DR HEDP group demonstrated 60% cohesive failures and 40% mixed failures. The saline group had 60% mixed failures and 40% adhesive failures. The representative images of the failure modes in different experimental groups are illustrated in Figs. 2 and 3 .

### FTIR Analysis

#### Amide III:Phosphate Ratio

Pretreatment ratio was higher with DR HEDP (16.4 ± 4.0) when compared with Triton (11.6 ± 1.2) ( *p* = 0.039) and saline (11.4 ± 2.0) ( *p* = 0.031). Between Triton and saline, there was no significant difference ( *p* = 0.989). Posttreatment ratio demonstrated a significant increase for saline (28.4 ± 4.0) group, when compared with DR HEDP (12.7 ± 3.4) ( *p* < 0.001) and Triton (8.6 ± 0.6) ( *p* < 0.001). There was no significant difference between DR HEDP and Triton groups ( *p* = 0.131) ( Table 1 ).

**Table 1:** Mean and standard deviation values (multiplied by 1,000) for the ratios of amide III:phosphate and carbonate:phosphate on root dentin surface before (pre) and after (post) treatment (30 minutes) with different irrigants

Experimental groups	Amide III/phosphate (pre)	Amide III/phosphate (post)	Carbonate/phosphate (pre)	Carbonate/phosphate (post)
Triton	0.0117 ± 0.0012 ^a^	0.0086 ± 0.0007 ^a^	0.0257 ± 0.0028 ^a^	0.0281 ± 0.0036 ^a^
DR HEDP	0.0165 ± 0.0040 ^b^	0.0127 ± 0.0034 ^a^	0.0459 ± 0.0067 ^b^	0.0396 ± 0.0034 ^b^
Saline (control)	0.0115 ± 0.0020 ^a^	0.0285 ± 0.0040 ^b^	0.0371 ± 0.0059 ^b^	0.0327 ± 0.0029 ^a^

Abbreviations: ANOVA, analysis of variance; DR HEDP, Dual Rinse 1-hydroxyethylidene-1,1-diphosphonic acid.

Note: Different lowercase letters indicate statistically significant intergroup differences (one-way ANOVA with the post hoc Tukey's honest significant difference test).

#### Carbonate:Phosphate Ratio

**Fig. 4:**
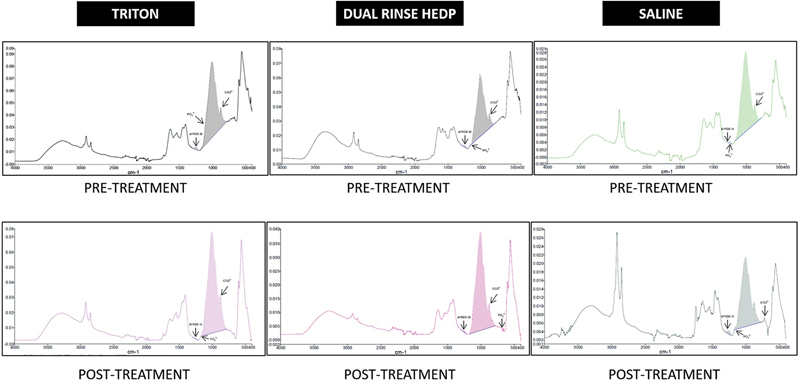
Superimposed infrared spectra of root dentin subjected to pre- and postirrigation with DR HEDP, Triton, and saline.

Pretreatment ratio was lower in Triton group (25.6 ± 2.7) when compared with DR HEDP (45.8 ± 6.7) ( *p* < 0.001) and saline groups (37.1 ± 5.9) ( *p* = 0.015). There was no difference between the DR HEDP and saline groups ( *p* = 0.062). The posttreatment ratio was higher in the DR HEDP (39.6 ± 3.4) group compared with the Triton (28.1 ± 3.5) ( *p* < 0.001) and saline groups (32.7 ± 2.8) ( *p* = 0.016). There was no significant difference between the Triton and saline groups ( *p* = 0.109) ( Table 1 ). The absorbance spectrum of dentin treated with different irrigating agents is represented in Fig. 4 .

## Discussion

For the optimal efficacy of root canal sealers, matching the irrigation protocol to the obturation strategy is important. 24 The current study investigated the POBS of CeraSeal and AH Plus sealers to root dentin conditioned with two different all-in-one root canal irrigants. Since the tested irrigants had different impact on the POBS of sealers tested, the null hypothesis was rejected. The results of the current study demonstrated that DR HEDP group had better bond strength than Triton for both CeraSeal and AH Plus sealers. This outcome is consistent with previous literature that has demonstrated the beneficial effect of DR HEDP on the adhesion of both CaSi- 11 and epoxy resin-based sealers. 11 33 The superior performance DR HEDP, with respect to dislodgement resistance of CeraSeal and AH Plus sealers is in line with the results obtained with the FTIR analysis performed in this study. DR HEDP significantly increased both amide III:phosphate and the carbonate:Phosphate as opposed to Triton, indicating its minimal damage to the root dentin. Previous research has reported that dentin treated with NaOCl/DR HEDP resulted in partially degraded, yet mineralized collagen fibers, with minimal alteration to the subsurface matrix. 34 This would have provided an ideal surface for the hydraulic CaSi sealer under investigation (CeraSeal) to micromechanically bond thereby yielding a higher bond strength to root canal dentin. It has also been reported that the mild chelating action rendered by DR HEDP allows in the reduction of the smear layer content thereby exposing the amino group in the dentinal collagen. This forms the covalent bond with the epoxy group of AH Plus sealer, thus improving its bond strength to root dentin. 35 Contrary to the results of the present study, one of the previous studies 33 reported that DR HEDP had lower POBS with respect to CaSi sealer (TotalFill BC sealer). The difference in these outcomes may be ascribed to the type of CaSi sealer used, irrigation technique, volume of the irrigants, and the technique of root canal obturation employed.

In the present study, samples treated with Triton demonstrated poor POBS with respect to both CeraSeal and AH Plus sealers. This might be due to two reasons. First, the presence of citric acid, which is a strong decalcifying agent when compared with DR HEDP. 36 37 Citric acid has been shown to demineralize and dissolve the inorganic content of root dentin more aggressively. 38 This might have resulted in poor POBS of CeraSeal sealer tested in the present study. 39 Second, the higher concentrations of NaOCl (<10%) present in Triton when compared with DR HEDP (3%) would have oxidized the organic matrix and deproteinized dentin collagen to a greater extent. 40 This must have resulted in poor POBS of AH Plus sealer to dentin. Saline, which was used as a control, demonstrated the lowest POBS for both resin as well as CaSi sealers tested in the present study. This result aligns with the previous similar studies 11 41 and can be attributed to its inability to dissolve organic as well as inorganic components of the dentin and thus compromising the bond strength of sealers.

The current study evaluated the structural changes of dentin matrix treated with experimental irrigants using ATR-FTIR, since this technique is commonly employed by numerous previous studies. 11 42 The dentinal samples were exposed to experimental irrigants for 30 minutes. This time period was chosen as per the previous study, which demonstrated that a minimum of 30-minute exposure time of the irrigation solutions was required to distinctly evaluate the dentinal changes in the amide III:phosphate and carbonate:phosphate ratios. However, for evaluating the POBS of CeraSeal and AH Plus sealers, the root canals were irrigated with the experimental irrigants for a shorter time period to mimic the clinical scenario. 32 This could be one of the constraints of the current study. Nevertheless, FTIR does give some information regarding the changes observed in the organic and inorganic content of the dentin matrix caused by decalcifying and deproteinizing agents used.

POBS is a commonly used technique to assess the adhesion of endodontic sealers to the radicular dentin. Its reliability in measuring the marginal adaptability of the root filling material to the surrounding dentin has been well documented in previous studies. 11 43 To standardize the POBS technique, it is advised that the ratio of the plunger's diameter to the specimen's diameter be maintained between 0.6 and 0.85, 44 which was implemented in the current study. Fractographic analysis was performed to test the type of bond failures after the POBS analysis. For both AH Plus and CeraSeal sealers, samples treated with DR HEDP had maximum cohesive failures when compared with Triton, reflecting its beneficial effects on the bonding of both the sealers to root dentin. Saline, which was used as a control group, had maximum adhesive failures with respect to both sealers, owing to its poor root dentin conditioning effect, leading to compromised bond strength to sealers.

Temperature conditions are known to affect the FTIR analysis by influencing their molecular behavior, evaporation, and instrumental sensitivity. It can lead to shifts in vibrational frequencies, changes in the intensity of absorption peaks, and variations in the overall spectrum. Hence, a standard room temperature condition of 23°C was maintained throughout the experimental procedure, which was monitored using a digital thermometer.

This research remains restricted owing to its *in vitro* methodology. The POBS measurement obtained in this study may vary in clinical scenarios due to the variations in the irrigation techniques, nature of the dentin, canal anatomy, root filling technique, and moisture present in the root canal. Hence, further clinical studies are warranted to evaluate the effect of Triton and DR HEDP irrigants on the longevity of root-filled teeth obturated with AH Plus or CaSi sealers.

## Conclusion

In conclusion, DR HEDP exhibited the highest dislodgement resistance of both AH Plus and CeraSeal sealers to root dentin when compared with Triton and saline. Saline demonstrated the lowest dislodgement resistance with respect to both sealers tested. Posttreatment organic and inorganic content was significantly increased with DR HEDP.
